# Upper Limb Ischemic Gangrene as a Complication of Hemodialysis Access

**DOI:** 10.1155/2015/830219

**Published:** 2015-02-25

**Authors:** Shamir O. Cawich, Emil Mohammed, Marlon Mencia, Vijay Naraynsingh

**Affiliations:** Department of Clinical Surgical Sciences, University of the West Indies, St. Augustine Campus, St. Augustine, Trinidad and Tobago

## Abstract

Upper limb ischemia is a well-recognized complication of dialysis access creation but progression to gangrene is uncommon. We report a case of upper limb ischemic gangrene and discuss the lessons learned during the management of this case. Clinicians must be vigilant for this complication and they should be reminded that it requires *urgent* management to prevent tissue loss.

## 1. Introduction

Hemodialysis is the commonest form of renal replacement therapy for patients with chronic kidney disease in the Caribbean [1, 2]. With an increasing number of persons being diagnosed with chronic kidney disease and more hemodialysis accesses being created, we have noticed a concomitant rise in access-related complications [3, 4].

We report the case of a patient with a steal syndrome that was neglected until there was gangrene requiring amputation. Our message is to remind clinicians who encounter patients with hemodialysis accesses that a steal syndrome is a complication that requires emergent intervention to avert the threat of limb loss.

## 2. Presentation of a Case

A 58-year-old woman with diabetes mellitus and stage V chronic kidney disease received maintenance hemodialysis through a brachioaxillary PTFE graft in the left upper limb. The graft was in use for three years prior to presentation.

Approximately one week prior to presentation, graft thrombosis developed. She sought attention at a facility in North America where her original graft was implanted. Reportedly, significant thrombus load was evacuated and a self-expanding metallic stent was placed across a stenosed area near the arterial anastomosis. The procedure was reported to be uneventful, although technical details and stent size were not reported. She was discharged within 24 hours and returned to her country of residence in the Caribbean.

**Figure 1:**
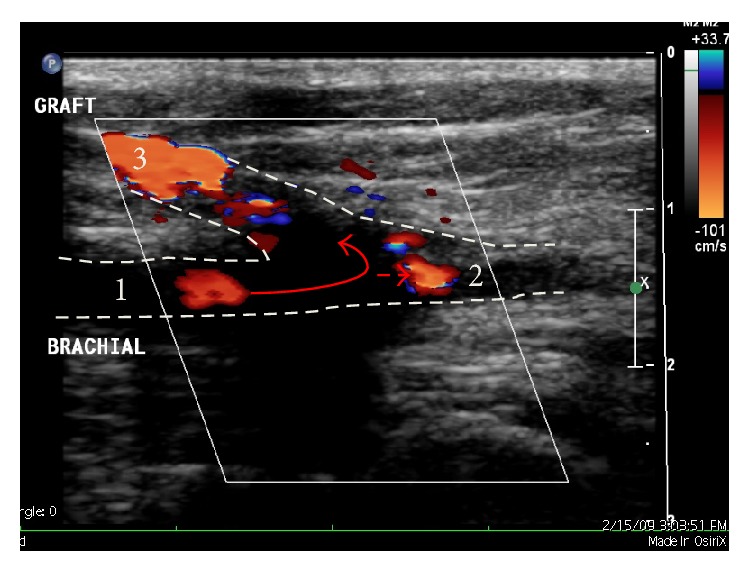
Duplex Doppler ultrasound of the left antecubital fossa demonstrating a significant steal syndrome. Blood enters the proximal brachial artery (1) and >70% is shunted through the PTFE graft (3) with <30% flow through the native distal artery (2).

Within 36 hours, she presented to the emergency room complaining of persistent pain in the ipsilateral upper limb, affecting mostly the fingers and hand. The pain was associated with numbness in the fingers and exacerbated by hand movements. She was evaluated by the emergency room physician and discharged with an increased analgesic prescription. She returned to the emergency room on two further occasions with similar complaints. At her last presentation, the emergency physician detected a thrill in the graft but the radial and ulnar pulses were weak. Therefore, a duplex Doppler ultrasound was ordered and suggested the presence of a steal syndrome (Figure 1). She was referred to the surgical team on call.

**Figure 2:**
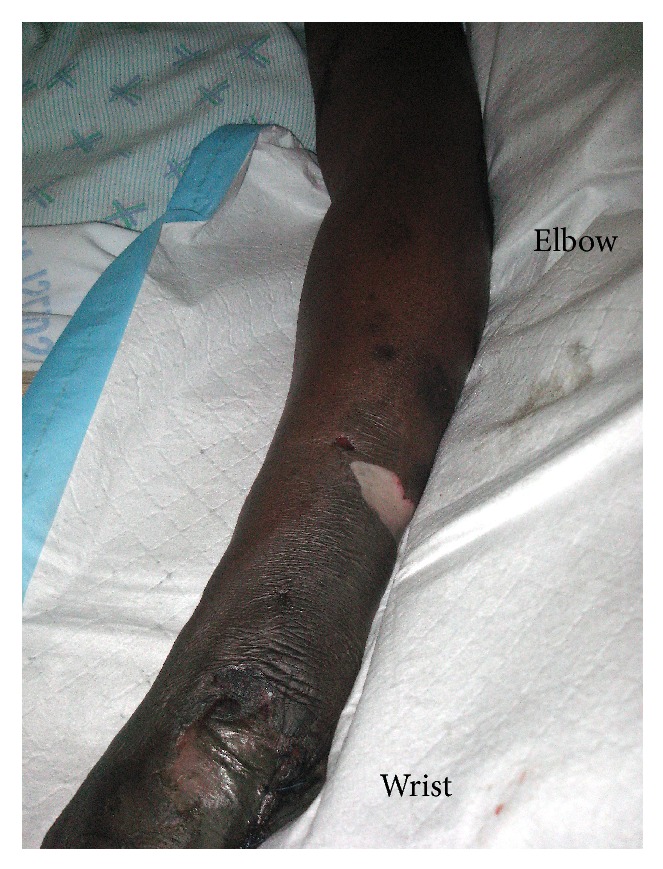
Clinical photograph of the left upper limb of a patient with dry gangrene to the midforearm level. Note the presence of blebs in the midforearm and distal forearm.

Upon assessment by the surgeons, she was noted to be in severe pain. Dry gangrene was already present at the ipsilateral limb extending up to the distal third of the forearm (Figure 2). There was a strong thrill and bruit at the graft but the pulses at the wrist were absent. As there was no vascular surgeon at the facility in which she was admitted, she was air-lifted to our service for definitive management.

**Figure 3:**
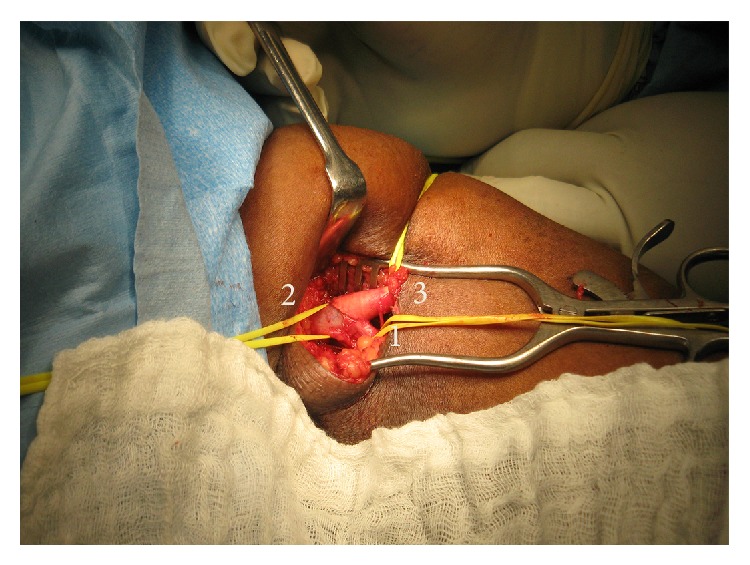
Operative photograph of the dissection in the antecubital fossa demonstrating the proximal (1) and distal (2) brachial artery. The anastomosis (3) is seen clearly and the PTFE graft is seen coursing proximally to the axillary vein.

**Figure 4:**
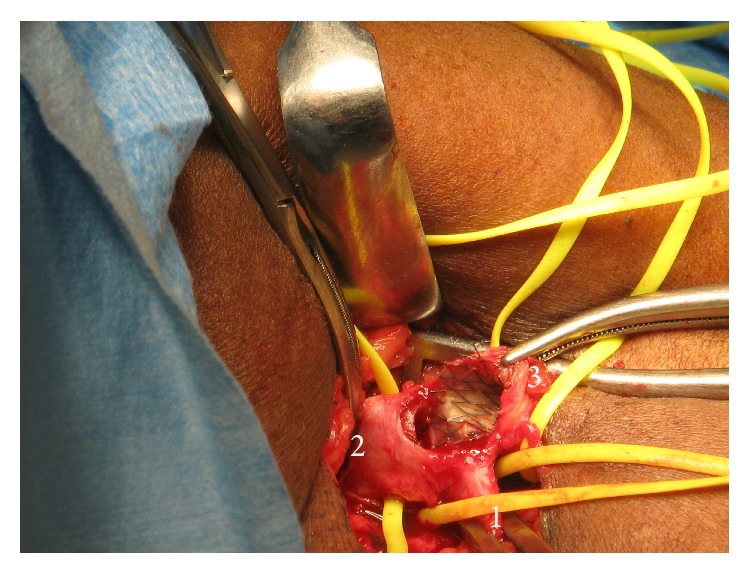
Operative photograph showing the proximal (1) and distal (2) native brachial artery. The anastomosis has been opened and reveals the metallic stent traversing the anastomosis and coursing up the graft (3). This required excision of the anastomosis and repair with a PTFE cuff.

**Figure 5:**
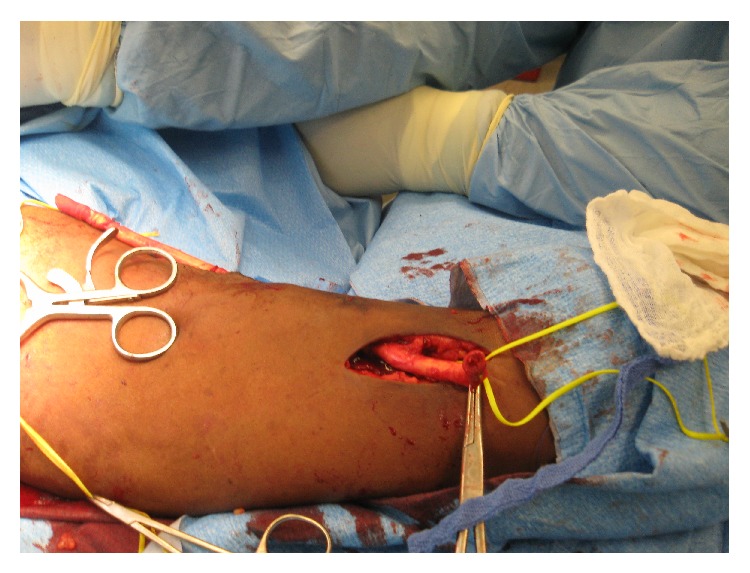
The anastomosis has been excised completely and the remnant defect repaired. The graft is being excised from the upper limb.

**Figure 6:**
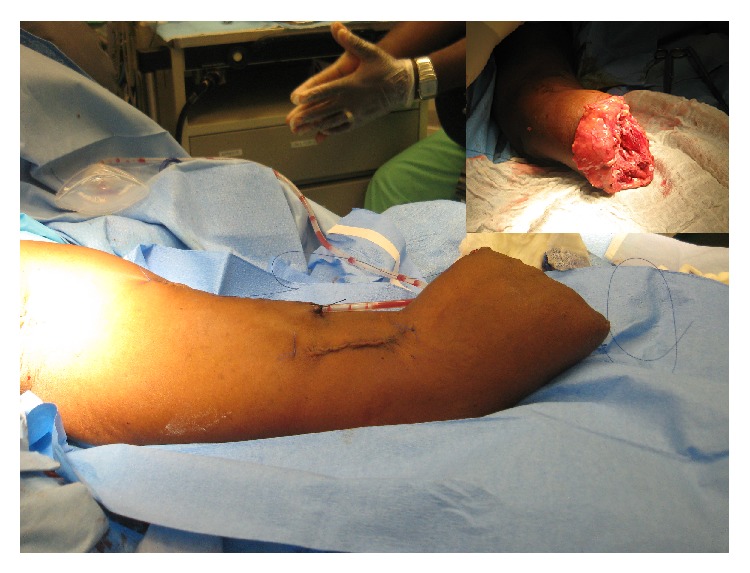
Viable tissue was only present at the proximal third of the forearm (inset). An amputation at the proximal third of the forearm was required.

She was taken immediately to the operating room where an incision was made over the arterial anastomosis in the left antecubital fossa. The anastomotic site was identified in preparation for access ligation (Figure 3). We attempted ligation by leaving a 1 cm cuff of PTFE graft distal to the anastomotic site but this was not feasible due to the presence of the metallic stent (Figure 4). The anastomosis was taken down, PTFE and metallic stent were excised completely, and the native vessel was repaired with a patch (Figure 5). The forearm was turgid and gangrenous. Therefore, we proceeded with a midforearm amputation at the same sitting (Figure 6). A temporary catheter was also introduced into the right internal jugular vein for continued hemodialysis.

Postoperatively, she had a prolonged recovery but the wounds healed uneventfully with no further wound-related complications.

## 3. Discussion

Storey et al. [5] was the first to report a “steal syndrome” after upper limb hemodialysis access creation in 1969. It is now a well-recognized complication of hemodialysis accesses, but the progression to upper limb gangrene is uncommon. On review of the literature, we only encountered 11 reports of upper limb gangrene related to hemodialysis accesses [6–15]. Once gangrene develops, an amputation is inevitable and brings associated morbidity and increased mortality. Therefore, upper limb ischemia should be considered a serious complication and treated with urgency to avert the threat of limb loss.

The literature contains many reports of steal syndromes after access creation. Davidson et al. [16] proposed a standardized clinical definition characterized by persistent severe ischemic symptoms (pain or weakness) distal to the access with a temporal relationship to access creation or manipulation. Using this definition, the incidence of steal syndrome varies from 1.6% [17] to 6.2% [16].

Many have explored the factors that may predict a risk of developing the steal syndrome. The presence of diabetes mellitus seems to be a strong predictor [5–7, 16, 18], with incidence of steal syndrome ranging from 5.5% [19] to 6.2% [16] in persons with diabetes. Other risk factors include increased age [7, 16, 20], female gender [17], smoking [17, 21], presence of peripheral arterial occlusive disease [7], use of PTFE grafts [17], and proximal access sites [17]. Several of these factors were present in our patient.

There is a wide variation of symptomatology in these patients, with the clinical manifestations allowing disease categorization into four stages [22]. In stage I there are pallor, cyanosis, and decreased temperature of the fingers and palm and in stage II there is pain only during hemodialysis; stage III indicates pain at rest and stage IV indicates the presence of ischemic ulcers, apical necrosis, and/or gangrene. Our patient presented initially with stage III disease but delayed intervention allowed rapid progression to stage IV. This reinforces the need for vigilant surveillance in patients who have had access creation and/or manipulation.

Although this is usually a clinical diagnosis made on history and examination, duplex Doppler ultrasound may provide confirmation when the classic signs are present: reduced pulse volume distal to the access, flow inversion at the anastomosis site, monophasic distal flow, arterial pressures <50 mmHg, and wrist-brachial index <0.4 [23]. Although the Doppler in this case was clearly suggestive of a steal syndrome, there was a disappointing lack of urgency in treatment, highlighting the need to remind clinicians of this diagnosis and the appropriate therapeutic regimes.

In stages I and II disease, medical management is an option using combinations of aspirin, clopidogrel, calcium antagonists, Pentoxifylline, naftidrofuryl, peripheral vasodilators, and anticoagulation [23]. Close surveillance is still mandatory because the disease will progress in up to 33% of patients despite medical management [23, 24].

In this case, however, immediate operative intervention would have been more appropriate. Operative intervention has two aims: the priority is to increase flow through the forearm arteries and a secondary goal is to maintain sufficient flow through the access to maintain dialysis. Several operative options would have been available.

### 3.1. Access Ligation

Access ligation can rapidly correct limb ischemia [22, 25, 26] and provides the greatest chance of limb salvage but sacrifices the access. This is usually reserved for patients who have impending tissue loss in stage III or stage IV disease. Access ligation was eventually performed in this case in an attempt to limit the rapid progression of ischemic necrosis but was too late to preserve the limb.

### 3.2. Access Restriction

Access banding refers to a procedure that limits flow through the access by reducing the diameter of the conduit just distal to the anastomosis. The resultant increased resistance redirects flow into the forearm vessels. This, however, has been greeted with inconsistent results because it is difficult to standardize the restriction created [27]. Some have advocated a modified banding technique known as the MILLER (Minimally Invasive Limited Ligation Endoluminal Revision) procedure to create a standardized restriction [28, 29]. This involves the use of a balloon catheter that is inflated just distal to the anastomosis until there is 60–80% reduction in luminal diameter at the access limb [30]. The balloon is left inflated and the access limb is dissected to allow a polypropylene suture to be passed 360° around the access and tied over the balloon, thereby restricting access inflow. Miller et al. [30] studied a cohort of 183 patients with steal syndromes and reported 89% technical success after the initial MILLER banding and 96% success with repeated bandings. This resulted in 75% primary patency at 6 months and 89% secondary patency at 24 months. Zangan and van Ha [28] described a similar technique using an external ligature over a constrained stent within the graft lumen to reduce the inflow diameter to 4 mm.

### 3.3. Access Revision

Distal Revascularization and Interval Ligation (DRIL) was initially described by Schanzer et al. in 1988 [31] on three patients with steal syndromes. The DRIL procedure aims to preserve the access but increase distal flow in the limb. This is achieved with an arterial bypass from the brachial artery at least 7 cm proximal to the access jumped into the artery just distal to the access anastomosis. The native artery is then ligated just distal to the access. By creating a low resistance conduit proximal to the access anastomosis with a concomitant relative increase in the resistance in the access limb due to ligation, there is a change in hemodynamics with preferential flow down the lower resistance bypass limb.

Schanzer et al. [14] reported 96% access patency and 100% symptom relief after 2 years in 23 patients post-DRIL procedures. The largest series to date was reported by Huber et al. [32] who performed 64 DRIL procedures. They reported 78% symptom relief, 77% primary patency, and 81% secondary patency at one year. Others have reported up to 90% ischemic ulcer healing rates after DRIL procedures [27].

An alternative to interrupting the native brachial artery is to perform a Revision Using Distal Inflow (RUDI) procedure as described by Minion et al. [33] in 2005. Here, the access is ligated just distal to the anastomosis and a new anastomosis is created using a smaller artery more distal in the limb. The RUDI procedure leaves intact flow in at least one forearm vessel, thereby increasing flow to the hand while maintaining access patency [33].

The final surgical option is Proximalization of Arterial Inflow (PAI) [24]. Here the existing access is taken down and a new one is created using a proximal artery, commonly the axillary artery [20]. There are two theories explaining how PAI works [20]. Firstly, there is increased pressure at the split point between the arm and the access (because the access takes blood from a higher-flow vessel), thereby leaving an increased amount of flow to descend in the normal vasculature. Secondly, with a higher access takeoff there will be a greater propensity for collaterals to form, taking advantage of the scapular anastomoses.

There are several operative options available to restore arterial flow to the limb threatened by a steal syndrome, but the majority of experience exists between the DRIL and MILLER procedures, which have comparable intermediate term results. There is ongoing debate about the indications and it is still unsettled which of these should be the first line procedure.

## 4. Conclusion

Although upper limb ischemia is a well-recognized complication of dialysis access creation, there are few reports of gangrene as a result of ischemia. Clinicians must be vigilant for this complication and they should be reminded that it requires emergent management to prevent tissue loss.

In the long term, dialysis patients should be monitored closely to identify potential graft complications that may require intervention. This must be facilitated by free communication between nephrologists, interventional radiologists, and vascular surgeons in the management of these cases.
